# Construction of a B cell-related gene pairs signature for predicting prognosis and immunotherapeutic response in non-small cell lung cancer

**DOI:** 10.3389/fimmu.2022.989968

**Published:** 2022-10-27

**Authors:** Xuanzong Li, Ruozheng Wang, Shijiang Wang, Linlin Wang, Jinming Yu

**Affiliations:** ^1^ Department of Radiation Oncology, Shandong Cancer Hospital and Institute, Shandong First Medical University and Shandong Academy of Medical Sciences, Jinan, China; ^2^ Department of Radiation Oncology, Affiliated Tumor Hospital of Xinjiang Medical University, Urumqi, China; ^3^ Department of Radiation Oncology and Shandong Provincial Key Laboratory of Radiation Oncology, Shandong Cancer Hospital and Institute, Shandong First Medical University and Shandong Academy of Medical Sciences, Jinan, China; ^4^ Research Unit of Radiation Oncology, Chinese Academy of Medical Sciences, Jinan, China

**Keywords:** non-small cell lung cancer, B cell marker genes, prognostic signature, immunotherapy, gene repair

## Abstract

**Background:**

Accumulating evidence indicates that the B cells play important roles in anti-tumor immunity and shaping tumor development. This study aimed to explore the expression profiles of B cell marker genes and construct a B cell-related gene pairs (BRGPs) signature associated with the prognosis and immunotherapeutic efficiency in non-small cell lung cancer (NSCLC) patients.

**Methods:**

B cell-related marker genes in NSCLC were identified using single-cell RNA sequencing data. TCGA and GEO datasets were utilized to identify the prognostic BRGPs based on a novel algorithm of cyclically single pairing along with a 0-or-1 matrix. BRGPs signature was then constructed using Lasso-Cox regression model. Its prognostic value, associated immunogenomic features, putative molecular mechanism and predictive ability to immunotherapy were investigated in NSCLC patients.

**Results:**

The BRGPs signature was composed of 23 BRGPs including 28 distinct B cell-related genes. This predictive signature demonstrated remarkable power in distinguishing good or poor prognosis and can serve as an independent prognostic factor for NSCLC patients in both training and validation cohorts. Furthermore, BRGPs signature was significantly associated with immune scores, tumor purity, clinicopathological characteristics and various tumor-infiltrating immune cells. Besides, we demonstrated that the tumor mutational burden scores and TIDE scores were positively correlated with the risk score of the model implying immune checkpoint blockade therapy may be more effective in NSCLC patients with high-risk scores.

**Conclusions:**

This novel BRGPs signature can be used to assess the prognosis of NSCLC patients and may be useful in guiding immune checkpoint inhibitor treatment in our clinical practice.

## Introduction

Lung cancer is one of the most common cancers in the world, with a high mortality rate (1). Non-small cell lung cancer (NSCLC) accounts for 80-85% of all lung cancers and mainly consists of lung adenocarcinoma (LUAD) and lung squamous cell carcinoma (LUSC) subtypes (2). Even though innovative treatment strategies, including immunotherapy and molecular targeted therapy, have revolutionized the management model of NSCLC patients, the 5-year overall survival (OS) rate for this population remains less than 20% (3). The reliable and clinically applied biomarkers for prognosis in NSCLC patients with all histological subtypes are still very rare. In addition, there are no well-established predictive biomarkers for immunotherapy response until now. Therefore, identifying reliable biomarkers to predict survival and guide appropriate personalized treatment for NSCLC patients is necessary and urgent in our clinical practice.

Accumulating evidence has revealed that tumor microenvironment (TME), accompanied by diverse tumor infiltrating lymphocytes (TILs), has been proven to play important roles in oncogenesis, tumor development and therapeutic efficacy prediction (4). In contrast to the well-investigated T cells (5), the potential role of tumor infiltrating B cells (TIL-B) is relatively less illustrated. Gottlin et al. found that the proliferative TIL-B could be identified in 35% of NSCLC, with significant variations in frequency across different clinical stages (6). B cells are a diverse population with highly heterogeneous subsets and functions (7). On the one hand, B cells can contribute to anti-tumor immunity by presenting antigens, producing antibodies, activating the complement cascade, assisting T-cell immune response, etc (8–10). On the other hand, there is also exist the regular B cells (Bregs) subset which can produce immunosuppression cytokines, such as IL-10 and IL-13, passively affects anti-tumor immunity (11). Recently, chen et al. demonstrated that the TIL-B has two major subtypes, namely the naïve-like and plasma-like B cells, with diverse functions in the progression of NSCLC (12). B cells were often associated with improved prognosis of NSCLC; however, the prognostic value of B cells is still controversial, with conflicting results across studies (13). Furthermore, several recent studies have found that B cells, associated mature tertiary lymphoid structures (TLSs) and plasma cells correlate with the efficacy of ICIs in multiple cancer types (14–19). Likewise, TLSs in tumors display substantial heterogeneity, and the prognostic and predictive value of TLSs is still controversial (20). However, a previous study demonstrated that only mature TLS with an active germinal center could predict the efficacy of immunotherapy in multiple cancer types (15). In fact, B cells are scarce in tumors without mature TLSs, whereas B cells are selectively activated and amplified in tumors with mature TLSs (21). Considering the significant roles of B cells in shaping the tumor immune environment and ICIs responses, therefore, it is necessary to make a comprehensive analysis of the heterogeneity, prognostic and immunotherapeutic predictive values of B cells in NSCLC.

Single-cell RNA-sequencing (scRNA-seq) method provides a potent approach for us to explore the complex biological behavior of TILs and potential mechanisms for them in shaping tumor development in various cancer types (22–24). Hence, establishing B cell-related signatures by means of scRNA-seq data could be a useful way to predict immunotherapeutic responses and prognosis in NSCLC patients. In this study, we successfully constructed a B cells-related gene pairs (BRGPs) prognostic signature in NSCLC utilizing the gene pair approach and data from scRNA-seq and bulk RNA-sequencing public datasets. Importantly, this novel BRGPs signature with no dependence upon specific gene expression levels can improve risk stratification, prognosis accuracy and individualized immunotherapy for NSCLC patients.

## Methods

### Data acquisition

**Figure 1:**
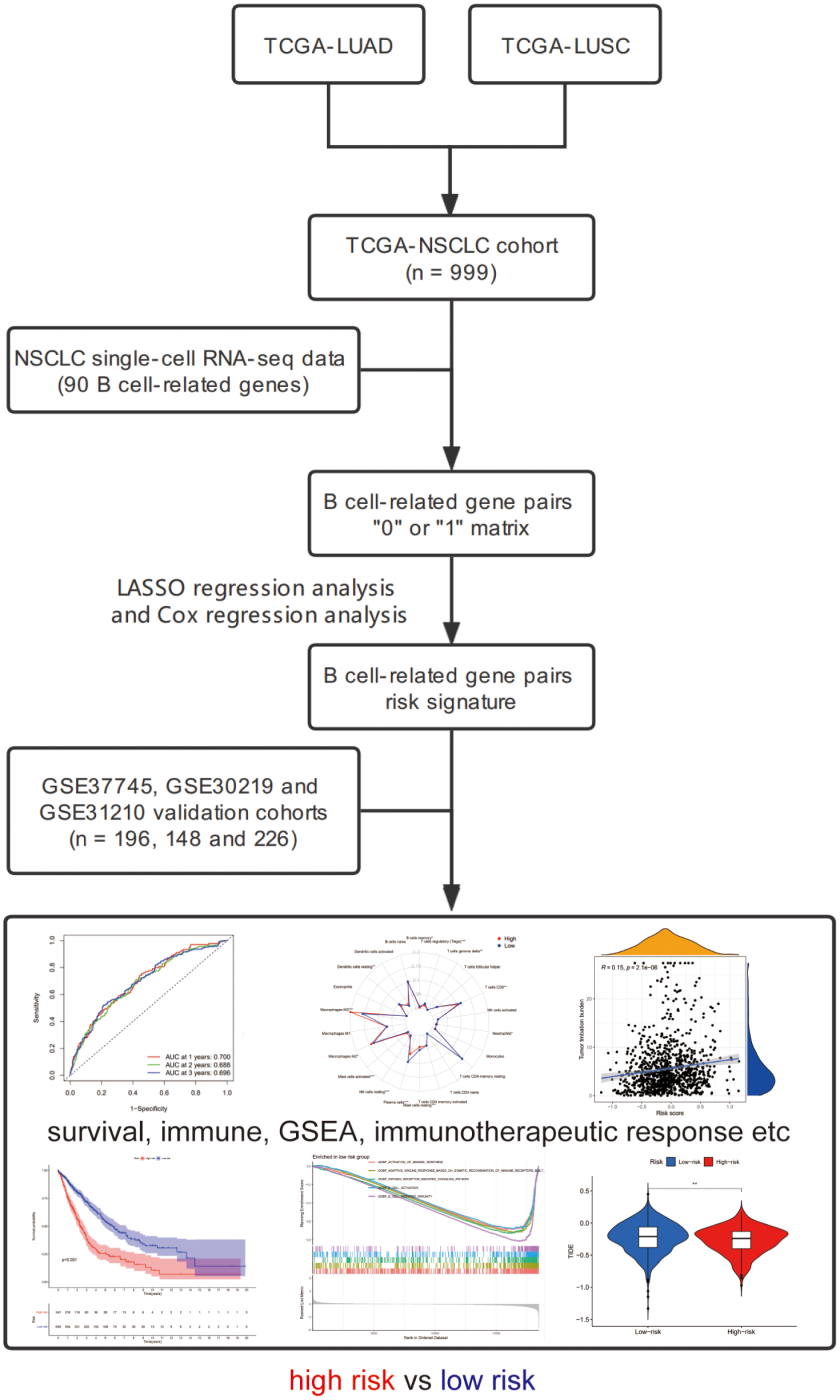
The flowchart of this study.

We downloaded the transcriptome sequencing data and corresponding clinical features of NSCLC (LUAD and LUSC) patients from TCGA website (https://portal.gdc.cancer.gov/) on September 2021. A total of 1016 cases with tumor or normal sequencing data were included in this cohort. We only selected tumor sequencing data to construct a gene signature. The merged TCGA-NSCLC dataset was regarded as the training cohort. Then, we employed three microarray datasets (GSE37745, GSE30219 and GSE31210) from GEO database and set it as the validation cohort (25). Finally, a total of 999 and 570 NSCLC patients harboring both available gene expression and corresponding clinical data were included in the training and validation cohorts, respectively. The flowchart of the present study design is shown in **Figure 1**.

### B cell-related genes used for analysis

A total of 22 cell clusters (C1-C22) and corresponding cluster-specific marker genes were retrieved from the additional files of one previous publication (12). Among these 22 cell clusters, C4 and C6 clusters were annotated as B cells, and their specific marker genes were utilized to be served as B cell-related genes (BRGs) (**Supplementary Table 1**). In detail, a total of 90 unique genes including 35 marker genes from C4 (naïve-like B cells) subset and 59 marker genes from C6 (plasma-like B cells) subset were defined as BRGs in this study.

### Identification of BRGPs in patients with NSCLC

BRGs were screened out using a median absolute deviation (MAD) >0.5, as those genes showed high variation in the samples from entire training cohort. Of note, these BRGs were also available in the validation cohort. Next, we used the gene expression levels of these BRGs in each sample for a pairwise comparison to construct BRGPs. For one BRGP (gene A|gene B), if the expression value of gene A was greater than gene B, the score of this pair was considered as 1. Otherwise, the score of gene A|gene B was defined as 0. The score of each BRGP in all samples were calculated, and those BRGPs with 1 or 0 less than 20% or more than 80% of total samples were excluded, since these pairs had low variation.

### Identification of prognostic BRGPs and construction of BRGPs signature

Using “survival” and “survminer” R packages, we performed univariate Cox regression analysis to identify prognostic BRGPs with the limitation condition for P value less than 0.05 in the training cohort. Subsequently, using “glmnet” R package, the least absolute shrinkage and selection operator (LASSO) regression analysis was conducted to reduce the number of BRGPs and avoid model overfitting. Finally, the multivariate Cox regression analysis was performed to calculate the coefficients of the remaining BRGPs and construct prognostic signature. The risk scores of BRGPs signature for each NSCLC patient were calculated based on the value of these BRGPs (0 or 1) in the signature and weighted by multivariate Cox regression coefficient. The formula was as follows: risk score = ∑βi ×(BRG A|BRG B)i, where β is the regression coefficient.

### Evaluation of prognostic capability of BRGPs signature in NSCLC patients

Using “survivalROC” R package, the 1-, 2-, and 3-year receiver operating characteristic (ROC) curve analyses were performed, and corresponding values of the area under the curve (AUC) were also calculated. The point of maximum Youden Index in the 3-year ROC curve was defined as the optimal cut-off point of the risk score (26, 27). The formula was as follows: YoudenIndex = Sensitivity+ Specificity-1. Based on the optimal cut-off value of BRGPs, NSCLC patients in training and validation cohorts were classified into high- and low-risk groups, respectively. The Kaplan–Meier method and log-rank test were applied to compare the survival curves of different risk groups. Then, the prognostic value of the risk score as well as other characteristics, including age, gender, histology and stage, were evaluated by univariate and multivariate Cox regression analysis. Furthermore, the association between BRGPs signature and these characteristics was analyzed by chi-square test, and the result was displayed by heatmap. Besides, the differences of the distribution of the risk scores in NSCLC patients with different TNM stages were also compared by Wilcoxon rank-sum test.

### Immune score, stromal score, tumor purity, and tumor-infiltrating analyses

Estimation of Stromal and Immune cells in Malignant Tumor tissues using Expression data (ESTIMATE) algorithm was employed to infer ESTIMATE, immune, and stromal scores and tumor purity based on “estimate” R package and gene transcriptional profiles (28). The distribution of the tumor purity, ESTIMATE, immune, and stromal scores were analyzed between high- and low- risk groups in NSCLC, respectively. Pearson correlation coefficient was used to compare the correlation relationships between the markers mentioned above and the risk score of BRGPs signature.

CIBERSORT algorithm as well as the LM22 gene signature were used to calculate the abundance of 22 different immune cell types in each tumor sample (29). Sample deconvolution was performed 1000 permutations and P < 0.05 was required. Wilcoxon rank-sum test was used to compare the proportions of each tumor infiltrate immune cell subsets between high- and low- risk groups. Then, the Kaplan–Meier method and log-rank test were used to evaluate the prognostic values of different tumor infiltrate immune cell subsets in NSCLC patients. In addition, xCell and MCP-counter algorithms were also used to calculate the abundance of different immune cell types in high- and low- risk groups (30, 31).

### Functional and pathway enrichment analyses

Gene set enrichment analysis (GSEA) was performed to functionally elucidate the biological roles of the BRGPs in NSCLC. Using the Gene Ontology (GO) gene set (c5.all.v7.4.symbols.gmt) and Kyoto Encyclopedia of Genes and Genomes (KEGG) gene set (c2.cp.kegg.v7.4.symbols.gmt) from the Molecular Signatures Database, we analyzed the signaling pathway enrichment status in NSCLC patients with high- and low-risk scores by GSEA. To achieve a normalized enrichment score for each analysis, gene set permutations with 1,000 times were carried out. A nominal P < 0.05 and false discovery rate (FDR) < 0.05 were regarded as significant results. Furthermore, we compared the enrichment levels of 29 immune-related functional signatures between high- and low-risk groups based on the single sample gene set enrichment analysis (ssGSEA) algorithm in the GSVA R package (32, 33).

### Prediction of immunotherapeutic response

The association between PD-L1 mRNA (CD274) expression and the risk scores was evaluated by Wilcoxon test and spearman correlation analysis. The gene mutation data of LUAD and LUSC patients were downloaded from TCGA database (https://portal.gdc.cancer.gov/), and the tumor mutational burden (TMB) scores of each NSCLC patient were calculated as mutations per million bases. Then, the distribution of TMB in high- and low-risk groups was compared by Wilcoxon test, and spearman correlation analysis were performed between the risk score and TMB. Moreover, the somatic mutation features of high- and low-risk groups were visualized in the waterfall plot by “maftool” R package in LUAD and LUSC patients, respectively. Tumor Immune Dysfunction and Exclusion (TIDE) algorithm has been proven to have robust power for predicting clinical responses of ICIs treatment in melanoma, NSCLC and other cancer patients (34). Using the TIDE web (http://tide.dfci.harvard.edu), we obtained TIDE score, T cell dysfunction score and T cell exclusion score, and the distribution of those scores in high- and low-risk groups were compared by Wilcoxon test, respectively.

### Statistical analysis

R software (version 4.1.1) was used to make all statistical analyses in this study, and P < 0.05 was considered statistically significant.

## Results

### Characteristics of patients with NSCLC in TCGA and GEO databases

A total of 999 patients from the TCGA-NSCLC dataset were defined as training cohort in this study. Besides, three independent NSCLC cohorts from GEO database were analyzed as the validation cohorts. The characteristics of the NSCLC patients in training and validation cohorts were provided in **Table 1**. Overall, in the training cohort, most patients over aged 65 years old (55.7%), were male (60.1%), had a disease stage I (54.4%), stage T2 (55.8%), stage N0 (64.2%), stage M0 (74.3%) and LUAD subtype (50.5%). Most patients with NSCLC in the GSE37745 cohort aged less than 65 years old (52.0%), were male (54.6%), in disease stage I (66.3%) and were LUAD (54.1%).

**Table 1:** Basic characteristics of the NSCLC patients from TCGA and GEO datasets.

Characteristic	TCGA-NSCLC	GSE37745	GSE30219	GSE31210
	(n = 999)	(n = 196)	(n = 148)	(n = 226)
Age (years), n (%)				
≤65	427 (42.7)	102 (52.0)	98 (66.2)	176 (77.9)
>65	556 (55.7)	94 (48.0)	50 (33.8)	50 (22.1)
Unknown	16 (1.6)	0	0	0
Gender, n (%)				
Female	399 (39.9)	89 (45.4)	25 (16.9)	121 (53.5)
Male	600 (60.1)	107 (54.6)	123 (83.1)	105 (46.5)
Disease stage, n (%)				
I	512 (51.3)	130 (66.3)	–	168 (74.3)
II	278 (27.8)	35 (17.9)	–	58 (25.7)
III	164 (16.4)	27 (13.8)	–	0
IV	33 (3.3)	4 (2.0)	–	0
Unknown	12 (1.2)	0	–	0
Histology, n (%)				
LUAD	504 (50.5)	106 (54.1)	85 (57.4)	–
LUSC	495 (49.5)	66 (33.7)	60 (40.5)	–
Other	0	24 (12.2)	3 (2.1)	–
T stage, n (%)				
T1	282 (28.2)	–	122 (82.4)	–
T2	557 (55.8)	–	18 (12.2)	–
T3	115 (11.5)	–	6 (4.1)	–
T4	42 (4.20%)	–	2 (1.3)	–
Unknown	3 (0.30%)	–		
N stage, n (%)				
N0	641 (64.2)	–	136 (91.9)	–
N1	222 (22.2)	–	12 (8.1)	–
N2	111 (11.1)	–	0	–
N3	7 (0.7)	–	0	–
Unknown	18 (1.8)	–	0	–
M stage, n (%)				
M0	742 (74.3)	–	148 (100)	–
M1	32 (3.2)	–	0	–
Unknown	225 (22.5)	–	0	–
EGFR/ALK mutation, n (%)				
Positive	103 (10.3)	–	–	138 (61.1)
Negative	439 (43.9)	–	–	88 (38.9)
Unknown	457 (45.7)	–	–	0

### Construction and validation of prognostic BRGPs signature

**Figure 2:**
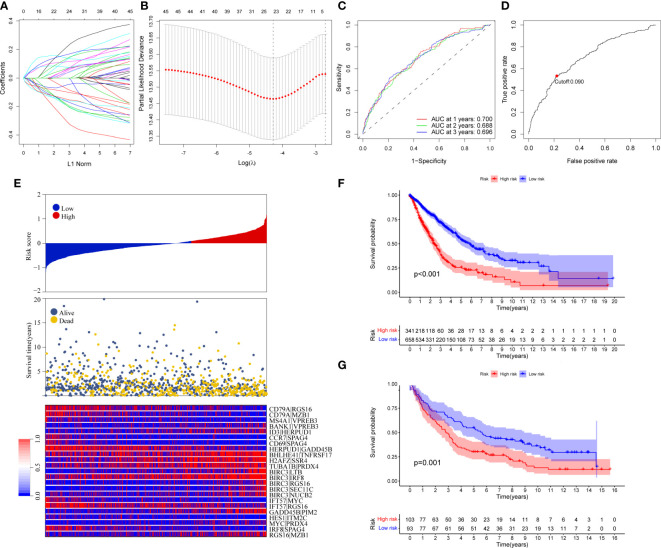
Construction and validation of BRGPs signature in NSCLC patients. **(A)** Trend graph of LASSO coefficients. **(B)** Partial likelihood deviation map. **(C)** ROC curve of 1-, 2- and 3-year survival predictions of BRGPs signature in the training cohort. **(D)** ROC curve of 3-year survival shows the optimal cut-off value of the risk score in the training cohort. **(E)** The distribution of BRGPs-based risk score, the vital statuses of patients and the heatmap of 23 BRGP profiles in the high- and low-risk groups. **(F, G)** Kaplan-Meier survival curves of OS in the training cohort **(F)** and validation cohort **(G)** based on risk score. BRGPs, B cell-related gene pairs; NSCLC, non-small cell lung cancer; ROC, receiver operating characteristic; OS, overall survival.

**Figure 3:**
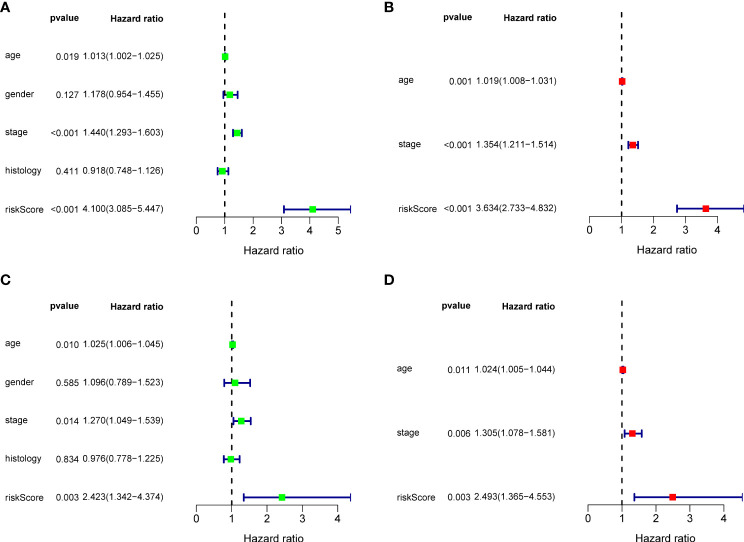
Cox regression analysis for BRGPs signature. **(A, B)** Forest plot of univariate **(A)** and multivariate **(B)** Cox regression analyses for the prognosis of NSCLC patients in the training cohort. **(C, D)** Forest plot of univariate **(C)** and multivariate **(D)** Cox regression analysis for the prognosis of NSCLC patients in the validation cohort. BRGPs, B cell-related gene pairs; NSCLC, non-small cell lung cancer.

Ninety unique B cell-related marker genes were included in this study, and 327 BRGPs with substantial variation was eventually identified using the method of cyclically single pairing along with a 0-or-1 matrix. In the training cohort, a total of 47 BRGPs had significant prognostic values. Lasso-penalized multivariate Cox proportional hazards modeling was performed on these prognostic BRGPs to improve stability and accuracy. After 1000 iterations, we successfully established a 23 BRGPs signature, consisting of 28 unique BRGs (**Figures 2A, B**). The detailed information of the 23 BRGPs signature was shown in **Table 2**. Besides, the expression levels of 28 unique BRGs were compared between NSCLC tumor and normal tissue in TCGA cohort, respectively. Importantly, most of BRGs (25/28) in BRGPs signature, except CCR7, HERUD1 and SEC11C, were differently expressed among NSCLC tumor and normal tissue (**Supplementary Figure 1**). We then calculated the risk score of BRGPs signature for each NSCLC patient in the training and validation cohorts. The 1-, 2-, and 3-year ROC curves were generated to assess the accuracy of BRGPs signature in predicting the prognosis of NSCLC patients. And the results revealed that this model was efficient in predicting the prognosis of NSCLC patients as AUC values were all around 0.700 (**Figure 2C**). In addition, the time-dependent ROC curve was applied to determine the optimal cut-off value for dividing patients into high- and low-risk subgroups (**Figure 2D**). Furthermore, for NSCLC patients in the training cohort, the risk score histogram, survival status distribution, and each corresponding BRGPs value were plotted (**Figure 2E**). Our results indicated that the BRGPs signature could efficiently distinguish good or poor survival of patients with NSCLC (P < 0.001) in the training cohort (**Figure 2F**). Importantly, univariate and multivariate Cox regression analyses demonstrated that the risk score of BRGPs signature was significantly associated with poor prognosis and could serve as an independent prognostic factor in NSCLC patients (**Figure 3A, B**). Furthermore, we verified the prognostic value of our prediction signature in the validation cohort. As expected, the results showed that low-risk patients had a significant longer OS compared with high-risk patients, either in GSE37745 (P = 0.001), GSE30219 (P = 0.027) and GSE31210 (P = 0.031) (**Figure 2G** and **Supplementary Figure 2**). And, the risk score was an independent prognostic factor based on univariate and multivariate Cox regression analyses in GSE37745 (**Figures 3C, D**).

**Table 2:** B cell-related gene pairs used for construction of prognostic risk model.

BRGP	BRG1	BRG2	Coefficient
CD79A|RGS16	CD79A	RGS16	-0.0481033
CD79A|MZB1	CD79A	MZB1	-0.1940487
MS4A1|VPREB3	MS4A1	VPREB3	-0.0761894
BANK1|VPREB3	BANK1	VPREB3	0.1293327
ID3|HERPUD1	ID3	HERPUD1	0.0590669
CCR7|SPAG4	CCR7	SPAG4	-0.0987176
CD69|SPAG4	CD69	SPAG4	-0.1857905
HERPUD1|GADD45B	HERPUD1	GADD45B	-0.1646793
BHLHE41|TNFRSF17	BHLHE41	TNFRSF17	0.035946
H2AFZ|SSR4	H2AFZ	SSR4	0.1132622
TUBA1B|PRDX4	TUBA1B	PRDX4	0.0671402
BIRC3|LTB	BIRC3	LTB	0.0749648
BIRC3|IRF8	BIRC3	IRF8	0.0622839
BIRC3|RGS16	BIRC3	RGS16	0.2188708
BIRC3|SEC11C	BIRC3	SEC11C	0.1388304
BIRC3|NUCB2	BIRC3	NUCB2	0.0500247
IFT57|MYC	IFT57	MYC	-0.149355
IFT57|RGS16	IFT57	RGS16	-0.1944459
GADD45B|PIM2	GADD45B	PIM2	0.1640718
HES1|ITM2C	HES1	ITM2C	-0.3062981
MYC|PRDX4	MYC	PRDX4	0.13737
IRF8|SPAG4	IRF8	SPAG4	-0.1565463
RGS16|MZB1	RGS16	MZB1	0.0361053

### Clinical significance of the BRGPs risk signature

**Figure 4:**
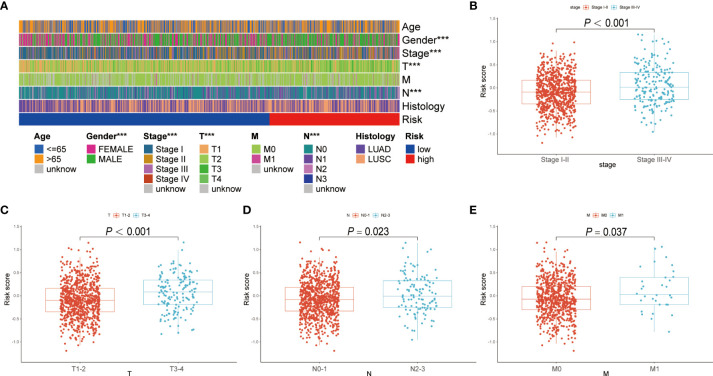
Correlation analysis between BRGPs signature and clinicopathological characteristics of NSCLC patients. **(A)** Heatmap reveals that patient’s gender (P<0.001), T stage (P<0.001) and N stage (P<0.001) are significantly related to BRGPs signature based on the chi-square test. **(B-E)** Box plot reveals that the risk scores of NSCLC patients are significantly related to the clinical stage **(B)**, T stage **(C)**, N stage **(D)**, and M stage **(E)** based on Wilcoxon rank-sum test. BRGPs, B cell-related gene pairs; NSCLC, non-small cell lung cancer. ***P < 0.001.

The correlation between the BRGPs signature and clinicopathological characteristics of NSCLC patients was estimated using a chi-square test. Gender (P < 0.001), disease stage (P < 0.001), T stage (P < 0.001) and N stage (P < 0.05) were found to be significantly related to BRGPs signature (**Figure 4A**). Furthermore, we used Wilcoxon rank-sum test and demonstrated that NSCLC patients with stage III-IV, stage T3-4, stage N2-3, and stage M1 had significantly higher risk scores than patients in stage I-II (P < 0.001), stage T1-2 (P < 0.001), stage N0-1 (P = 0.023), and stage M0 (P = 0.0037) (**Figures 4B–E**).

### Tumor immune microenvironment between high- and low-risk patients with NSCLC

**Figure 5:**
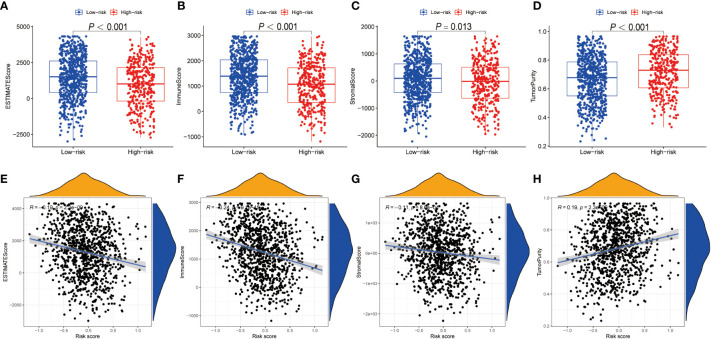
TME discrepancy between high- and low-risk groups. **(A–H)** The box plot shows the ESTIMATE score **(A)**, immune score **(B)**, stromal score **(C)** and tumor purity **(D)** of the high- and low-risk NSCLC patients. The correlation analysis between the ESTIMATE score **(E)**, immune score **(F)**, stromal score **(G)** as well as tumor purity **(H)** and the risk score in NSCLC patients, respectively. TME, tumor microenvironment; NSCLC, non-small cell lung cancer.

Using ESTIMATE algorithm, we evaluated the differences in immunologic landscapes between high- and low-risk NSCLC patients. The results showed that ESTIMATE score, immune score and stromal score were significantly higher in low-risk NSCLC patients compared with their counterparts (all P < 0.001) (**Figures 5A–C** **)**. By contrast, the tumor purity was significantly higher in the high-risk group (P < 0.001) (**Figure 5D**). Correspondingly, our findings suggested that ESTIMATE score, immune score and stromal score were all negatively correlated with the risk score (all P < 0.001) (**Figures 5E–G** **)**, whereas the tumor purity was positively correlated with the risk score (P < 0.001) (**Figure 5H**).

**Figure 6:**
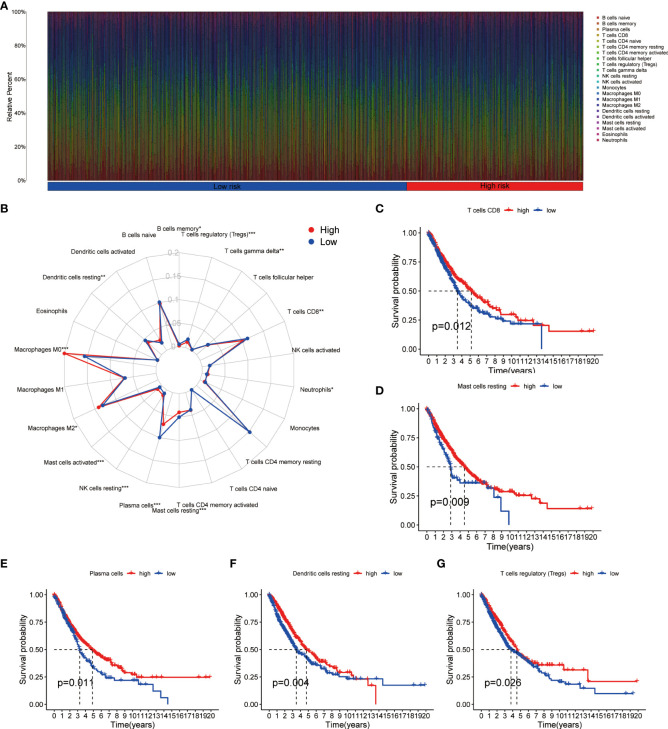
Immune cell infiltration analysis in NSCLC. **(A)** Relative infiltration proportions of 22 immune cells in each NSCLC sample based on CIBERSORT method. **(B)** The radar map reveals the distribution of 22 immune cells between high- and low-risk groups. **(C–G)** Comparison of overall survival for NSCLC patients with different infiltration levels of CD8+ T cells **(C)**, resting mast cells **(D)**, plasma cells **(E)**, resting dendritic cells **(F)** and regulatory T cells **(G)** in the training cohort, respectively. NSCLC, non-small cell lung cancer.

Using CIBERSORT method and LM22 single-cell gene expression model matrix, we compared the infiltration levels of 22 immune cells between high- and low-risk groups and the prognostic value of these immune cells in NSCLC patients. The relative expression landscape of these 22 immune cell types was described in each NSCLC patients (**Figure 6A**). We found that 12 immune cells were distributed with significant differences between the high- and low-risk groups (**Figure 6B**). Among these immune cells, the infiltration levels of CD8+ T cells, resting mast cells, plasma cells, resting dendritic cells, memory B cells, regulatory T cells (Tregs) and gamma delta T cells were higher in the low-risk group. Additionally, our findings indicated that CD8+ T cells, resting mast cells, plasma cells, resting dendritic cells and Tregs were all significantly associated with a favorable OS in patients with NSCLC (**Figures 6C–G**). On the contrary, the infiltration levels of neutrophils, resting NK cells, activated mast cells, M2 macrophages and M0 macrophages were higher in the high-risk group, and both were significantly associated with poor clinical outcomes in NSCLC patients (**Supplementary Figures 3A–E**). Furthermore, using additional immune deconvolution tools, we also demonstrated that several immune cells which primarily responsible for effective anti-tumor immunity, such as CD8+ T cell, CD4+ T cell and B-cells, were infiltrated higher in the low-risk group (**Supplementary Figures 4, 5**).

### Functional evaluation of the BRGPs signature

**Figure 7:**
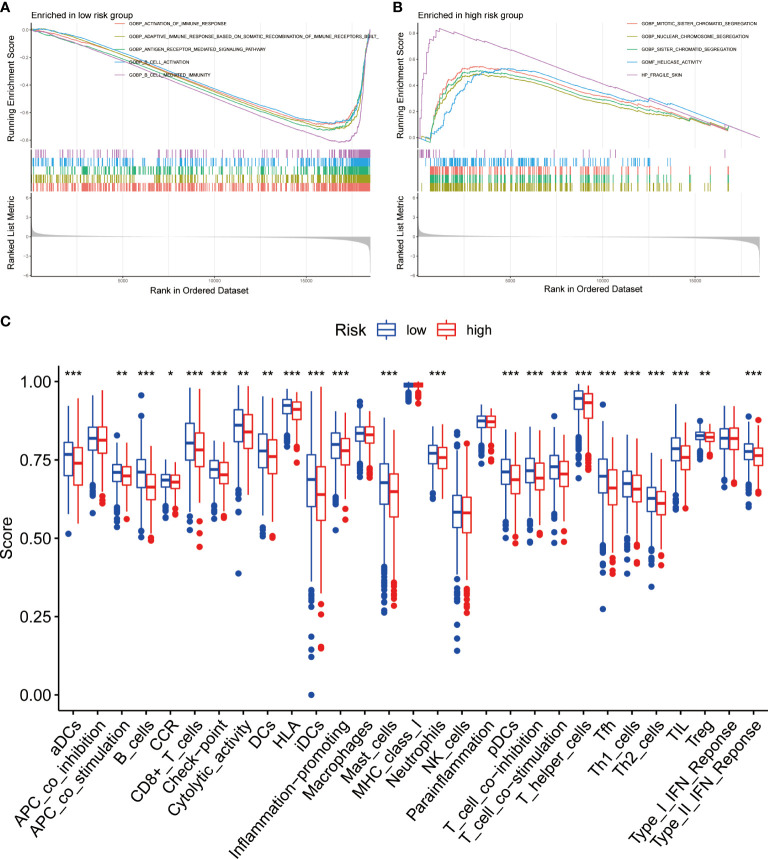
Function enrichment analysis of BRGPs signature in NSCLC. **(A, B)**. The GSEA analysis reveals the five most significant enrichment pathways in low- **(A)** and high-risk **(B)** NSCLC patients. **(C)** The ssGSEA analysis reveals that the relative enrichment score of 29 immune related signatures in NSCLC patients with high and low risk scores. BRGPs, B cell-related gene pairs; NSCLC, non-small cell lung cancer; GSEA, gene set enrichment analysis; ssGSEA, single sample gene set enrichment analysis. ***, P<0.001; **, P<0.01; *, P<0.05.

To identify the underlying biological characteristics on the basis of BRGPs signature, we performed GSEA to predict the most significant enrichment signaling pathways between high- and low-risk NSCLC patients. Our results suggested that patients with low-risk scores significantly enriched with several immune activation related pathways, including activation of immune response, adaptive immune response based on somatic recombination of immune receptors built from immunoglobulin superfamily domains, antigen receptor mediated signaling pathway, B cell activation and B cell mediated immunity (**Figure 7A**). Meanwhile, the pathways involved in cell proliferation, such as nuclear chromosome segregation, sister chromatid segregation, mitotic sister chromatid segregation and helicase activity, were maximum extent enriched in the high-risk group (**Figure 7B**). Likewise, KEGG analysis found that several immune activation related pathways, such as intestinal immune network for IgA production and B cell receptor signaling pathway were enriched in low-risk BRGPs subgroup (**Supplementary Figure 6**).

We assessed the expression profiles of 29 immune-associated features to determine their immune-related signaling pathways, cell types, and functional activities. We quantified the level of enrichment of 29 immune signatures in each NSCLC sample using ssGSEA method. We demonstrated that the immune-associated biological behavior of patients in high- and low-risk groups was significantly different. Notably, patients in the low-risk group scored significantly higher in most immune or inflammation-related pathways, except for APC_co_inhibition, macrophages, MHC_class_I, NK_cells, parainflammation and Type_I_IFN_Reponse (**Figure 7C**).

### BRGPs signature predicts immunotherapeutic response

**Figure 8:**
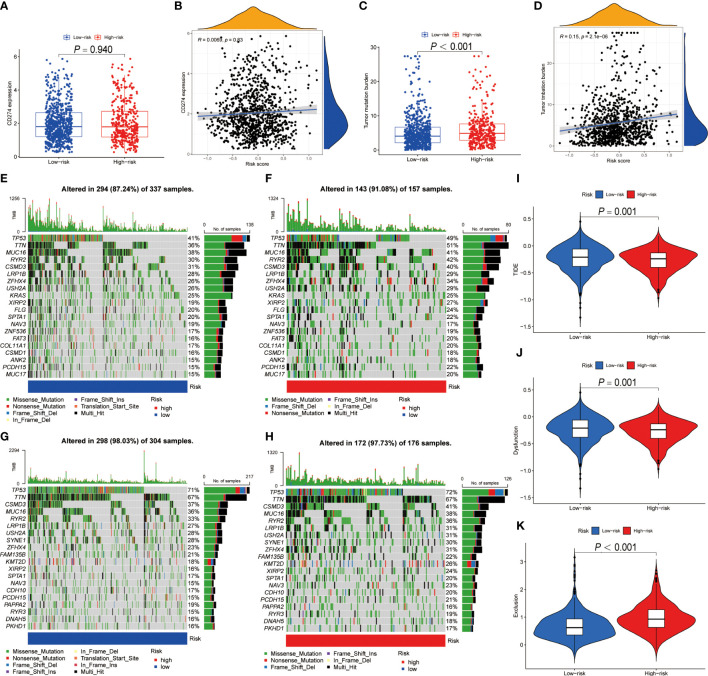
The predictive ability of BRGPs signature in NSCLC patients with immunotherapy. **(A–D)**. The box plot shows the PD-L1 mRNA (CD274) expression levels **(A)** and TMB scores **(C)** between high- and low-risk patients. The correlation analysis between the PD-L1 mRNA expression levels **(C)** and TMB scores **(D)** and the risk score in NSCLC patients, respectively. **(E–H)** The top 20 frequent mutation genes in low- **(E)** and high-risk **(F)** patients in TCGA-LUAD cohort. The top 20 frequent mutation genes in low- **(G)** and high-risk **(H)** patients in TCGA-LUSC cohort. **(I–K)** TIDE score **(I)**, T cell dysfunction score **(J)** and T cell exclusion score **(K)** between high- and low-risk patients. BRGPs, B cell-related gene pairs; NSCLC, non-small cell lung cancer; TMB, tumor mutational burden; LUAD, lung adenocarcinoma; LUSC, lung squamous cell carcinoma; TIDE, Tumor immune dysfunction and exclusion.

Numerous studies indicated that patients with high PD-L1 expression and TMB scores have a higher chance of benefiting from ICIs treatment (35–39). As a result, we evaluated the association between BRGPs signature and these two well-characterized immunotherapy biomarkers. Unfortunately, there was no significant difference in PD-L1 mRNA expression between high- and low-risk NSCLC patients (**Figure 8A**). Besides, our results indicated that the risk score of BRGPs signature was not correlated with PD-L1 mRNA expression levels in TCGA-NSCLC, GSE30219 and GSE37745, but was positively correlated with PD-L1 mRNA expression in GSE31210 (**Figure 8B** and **Supplementary Figure 7**). However, our findings indicated that NSCLC patients with high-risk score had significantly higher TMB scores (P < 0.001) (**Figure 8C**). Additionally, correlation analysis revealed a positive correlation between the risk score and TMB scores (P < 0.001) (**Figure 8D**). Moreover, the significant association between TMB score and the risk score of BRGPs signature was still existing in patients with either LUAD or LUSC (all P < 0.05) (**Supplementary Figures 8A, B**). Furthermore, the top 20 mutation genes of the high- and low-risk cohorts of LUAD and LUSC patients were plotted (**Figures 8E–H**). Then, we evaluated the relationship between BRGPs risk signature and TIDE-related scores. Interestingly, patients with high-risk scores had significantly higher exclusion scores, lower TIDE scores and lower T cell dysfunction scores compared with low-risk patients (all P < 0.01) (**Figures 8I–K**), implying that high-risk NSCLC patients may be more sensitive to immunotherapy. Unsurprisingly, an inferior survival rate for low-risk patients after immunotherapy were observed in GSE135222 (**Supplementary Figure 9**). Collectively, these findings indicate that patients with high-risk scores are more likely to benefit from immunotherapy and that BRGPs may serve as a potential biomarker for predicting immunotherapy efficacy in NSCLC patients.

## Discussion

The tremendous clinical success of cancer immunotherapy refocused attention on various TILs, however, reliable biomarkers based on the TILs to predict immunotherapy response and prognosis of NSCLC patients are still very rare (4). In this study, we obtained B cell specific marker genes from a scRNA-seq study and innovatively conducted a new method of cyclically single pairing along with a 0-or-1 matrix to construct a novel BRGPs signature in NSCLC patients. In the training and validation cohorts, our novel BRGPs signature demonstrated effective prognostic performance and can be used as an independent risk factor for NSCLC patients. Analysis of clinicopathological characteristics, TME conditions, immune profiles and biological pathway revealed that patients with a low-risk score were characterized by early clinical stage, low tumor purity, high anti-tumor immune cell infiltration and immune-active states. Additionally, we found that patients with high-risk scores had significantly higher TMB scores and lower TIDE scores compared with patients with low-risk scores, which indicates that high-risk patients are more likely to benefit from immunotherapy. Collectively, BRGPs signature might be a useful biomarker to predict prognosis and immunotherapeutic effect in NSCLC patients. More importantly, our novel BRGPs signature only needs to detect the higher or lower expression level of the two BRG in each BRGP without requiring quantitative gene expression profiles (40), which avoids potential technical bias and improves its clinical practicability.

In this study, the BRGPs signature was composed of 23 BRGPs, including 28 different BRGs. In the signature model, gene pairs (BIRC3|RGS16 and HES1|ITM2C) harbored the highest coefficients and presented positive and negative effects on the prognosis of NSCLC patients, respectively. BIRC3 acts as a member of inhibitors of apoptosis proteins (IAPs) family and plays an important role in pro-survival and antiapoptotic on the cells, which has been characterized in multiple cancer types (41). In LUAD, increased expression of BIRC3 could promote tumor growth and metastasis (42). RGS16 is one of the regulators of G protein singling (RGS) gene family members and negatively regulates G protein–coupled receptor (GPCR) signaling cascades (43). It was reported that RGS16 played central roles in immune and inflammatory responses (44, 45). Importantly, RGS16 can inhibit the Ras-Raf-MEK-Erk signaling cascade and promotes antitumor CD8+ T cell exhaustion (46). HES1, a Notch signaling pathway target, plays both oncogenic and tumor suppressor roles in different cell types (47). Interestingly, HES1, associated with Notch activation, was essential to inhibit the progression of B-cell acute lymphoblastic leukemia rather than T-cell acute lymphoblastic leukemia (47). Besides, HES1 has been shown to be positively correlated with the expression of FOXP3 and plays an important role in regulating the invasive and migratory functions of FOXP3 in NSCLC cells (48). ITM2C belongs to the Type II Integral Membrane protein (ITM2) family and is thought to be negatively regulates the amyloid-beta peptide production (49, 50). Importantly, ITM2C is highly and selectively expressed by Antibody Secreting Cells in the immune system (50). The signature genes identified in this study can provide potential targets for experimental design to give new insights into the pathological mechanisms in NSCLC.

We performed 1-, 2-, and 3-year ROC curves analysis to assess the efficacy and accuracy of the BRGPs signature, and the corresponding AUC values were all close to 0.700, indicating that our predictive signature was effective in predicting the prognosis of NSCLC patients. Zhang et al. identified a 13-gene B cell-associated signature in LUAD patients, with 2-year AUC of 0.621 in the training cohort, inferior to the AUCs in our study (51). Additionally, a previous study demonstrated significant differences in the expression levels of B cell-related genes between patients with LUSC who had a good survival outcome and those who had a poor survival outcome (52). In this study, the risk score of our BRGPs signature was an independent prognostic factor in NSCLC patients, and we found that the risk signature was significantly associated with the clinical stage of NSCLC patients. These findings revealed that the major clinical significance of the BRGPs signature and prompted us to explore the potential underlying mechanism.

Considering the remarkable impact of TME on the prognosis of cancer patients (53), we investigated the discrepancy in immune cell infiltration between low- and high-risk NSCLC patients. Notably, we found significant TME heterogeneity between high- and low-risk NSCLC patients using ESTIMATE and CIBERSORT methods. For example, our findings indicated that low-risk NSCLC patients had a higher proportion of CD8+ T cells, but a lower proportion of M2 macrophages. CD8+T cells have been linked to a better prognosis of patients with multiple cancer types (54). As the key effectors in the anti-tumor process, CD8+T cells can release perforin and granzyme and mediate cytotoxicity *via* Fas/FasL signaling pathway (55). Otherwise, the macrophages can be classified into M1 and M2 subtypes based on differentiation status and functional roles (54). Tumor-associated macrophages (TAMs) typically exhibit an M2-like phenotype which can secrete various immune suppress factors, including IL-10, TGFβ, and proangiogenic factors, and previous research has established a link between TAMs and disease progression and poor prognosis of NSCLC patients (56, 57). Then, the functional enrichment analysis revealed that immune-activating pathways were significantly enriched in low-risk NSCLC patients, whereas high-risk NSCLC patients were closely implicated in cell proliferation related functions. Indeed, several important BRGs found in BRGPs signature, such as BIRC3, IFT57, GADD45B and SPAG4, have been associated with the proliferation or migration of NSCLC cells (41, 42, 58–61). Therefore, high-risk NSCLC patients are more likely to harbor genome instability status and associated with high TMB, tumor progression, and relative advanced tumor stage. Collectively, BRGPs signature showed significant prognostic value in patients with NSCLC, and the potential biological mechanism may attribute to the dysregulation of the cell cycle and TME heterogeneity.

Currently, immunotherapy, especially for immune checkpoint blockade, has revolutionized the treatment of lung cancer (62). However, the response rate of ICIs is relatively low, and most NSCLC patients cannot benefit from these immunotherapeutic agents (63). Therefore, developing reliable biomarkers to improve the prognosis of NSCLC with ICIs treatment is urgently needed. Up to now, various biomarkers have been investigated to determine the therapeutic effect of ICIs (64, 65). For instance, PD-L1 expression and TMB scores have been demonstrated to be independently associated with the efficacy of ICIs and can be used to guide ICIs treatment in our clinical practice. Likewise, TIDE methods are widely used for immunotherapeutic prediction and have been proven to have impressive predictive performance in various cancers (34, 66–68). The relationship between above mentioned ICIs-related biomarkers and BRGPs signature was investigated in this study. Our findings indicated that TMB scores rather than PD-L1 mRNA expression were positively correlated with the risk score. In contrast to the unfavorable prognosis associated with high TMB scores in NSCLC patients (69), TMB is common positively correlated with the improved efficacy of immunotherapy (70). Unfortunately, a positive correlation between BRGPs signature and PD-L1 mRNA expression was not found in all NSCLC cohorts. Since PD-L1 tumor staining by immunohistochemical is routinely used as an immunotherapy biomarker in multiple cancer types including NSCLC, further studies to investigate the relationship between BRGPs signature and PD-L1 expression are urgently warranted both in the mRNA and protein levels. Importantly, we found that NSCLC patients with high risk-scores had significantly higher TMB scores but lower TIDE scores, implying a greater potential for immunotherapy benefit. Hence, ICIs treatment may be a better option for NSCLC patients with high-risk scores. Nevertheless, the predictive value of BRGPs serving as a reliable biomarker in immunotherapy requires further validation.

Undeniably, several limitations were existed in this study. Even though the prognostic value of our BRGPs signature was fully validated in TCGA and GEO cohorts, the study’ retrospective nature and the potential bias should not be neglected. Next, the results were achieved based on public database. Therefore, additional experimental studies (both *in vitro* and *in vivo*) are warranted to verify the molecular mechanism through which B cell-related genes affect NSCLC, and external clinical studies should be performed to further clarify the predictive capability of our BRGPs signature in NSCLC patients with and without immunotherapy.

In conclusion, we established a novel BRGPs signature that could serve as a potent prognostic biomarker and a potential indicator of immunotherapeutic response in NSCLC. Importantly, our BRGPs signature significantly correlated with TME and TMB, indicating that these molecular changes might explain the clinical significance. Nonetheless, future clinical studies will be required to validate the utility of the constructed BRGPs signature as soon as possible.

## Data availability statement

The datasets presented in this study can be found in online repositories. The names of the repository/repositories and accession number(s) can be found in the article/**Supplementary Material**.

## Author contributions

XL, SW and LW contributed to the study concept and design, and critical revision of the manuscript for important intellectual content. XL performed the data analysis and drafted the manuscript. All authors contributed to the article and approved the submitted version.

## Funding

This work was partially supported by funds from the Natural Science Foundation of Shandong Province (ZR2019LZL012, ZR201911040452), the Academic Promotion Program of Shandong First Medical University (2019ZL002), Research Unit of Radiation Oncology, Chinese Academy of Medical Sciences (2019RU071) and the foundation of National Natural Science Foundation of China (82172865, 81627901, 81972863 and 82030082).

## Acknowledgments

We would like to thank Freescience for English language editing.

## Conflict of interest

The authors declare that the research was conducted in the absence of any commercial or financial relationships that could be construed as a potential conflict of interest.

## Publisher’s note

All claims expressed in this article are solely those of the authors and do not necessarily represent those of their affiliated organizations, or those of the publisher, the editors and the reviewers. Any product that may be evaluated in this article, or claim that may be made by its manufacturer, is not guaranteed or endorsed by the publisher.
