# Supplementary material for: Construction of a B cell-related gene pairs signature for predicting prognosis and immunotherapeutic response in non-small cell lung cancer
Source: Front Immunol. 2022 Oct 27;13:989968. doi: 10.3389/fimmu.2022.989968 (PMC9647047; doi:10.3389/fimmu.2022.989968)
Supplement: Supplementary file 1 [file DataSheet_1.docx]

Supplementary Material

## Supplementary Table and Figures

**Supplementary Table 1.** B cell cluster specific marker genes identified in single-cell RNA-seq data.

| **Gene** | **cluster** | **Gene** | **cluster** | **Gene** | **cluster** |
| --- | --- | --- | --- | --- | --- |
| CD79A | C4 | IGHG3 | C6 | IGHV3-15 | C6 |
| MS4A1 | C4 | IGLC2 | C6 | IGHV1-69D | C6 |
| CD83 | C4 | IGHG2 | C6 | IGLV3-21 | C6 |
| BANK1 | C4 | IGKC | C6 | IGLV3-1 | C6 |
| ID3 | C4 | IGHA1 | C6 | IGLV2-11 | C6 |
| MEF2C | C4 | IGHG1 | C6 | IGHV3-33 | C6 |
| CCR7 | C4 | IGHG4 | C6 | IGHV1-18 | C6 |
| CD69 | C4 | JCHAIN | C6 | IGKV3-15 | C6 |
| CD74 | C4 | SSR4 | C6 | IGLV1-44 | C6 |
| TCF4 | C4 | MZB1 | C6 | IGKV3-20 | C6 |
| IGHM | C4 | FKBP11 | C6 | IGHV3-7 | C6 |
| HLA-DRA | C4 | DERL3 | C6 | IGHD | C6 |
| HERPUD1 | C4 | HERPUD1 | C6 | IGLV1-40 | C6 |
| BHLHE41 | C4 | PRDX4 | C6 | IGLV2-8 | C6 |
| IFI30 | C4 | IGHV3-30 | C6 | IGLV7-46 | C6 |
| H2AFZ | C4 | XBP1 | C6 | IGLV1-51 | C6 |
| TUBA1B | C4 | FKBP2 | C6 | IGKV1-39 | C6 |
| BIRC3 | C4 | CD79A | C6 | - | - |
| PHACTR1 | C4 | CD27 | C6 | - | - |
| IGHD | C4 | FAM92B | C6 | - | - |
| VPREB3 | C4 | SPAG4 | C6 | - | - |
| IFT57 | C4 | SEC11C | C6 | - | - |
| GADD45B | C4 | JSRP1 | C6 | - | - |
| PMAIP1 | C4 | ITM2C | C6 | - | - |
| SPIB | C4 | HSP90B1 | C6 | - | - |
| LINC01781 | C4 | NUCB2 | C6 | - | - |
| LTB | C4 | FCRL5 | C6 | - | - |
| HES1 | C4 | TNFRSF17 | C6 | - | - |
| TUBB4B | C4 | PIM2 | C6 | - | - |
| MYC | C4 | SDF2L1 | C6 | - | - |
| IRF8 | C4 | IGLC3 | C6 | - | - |
| RGS16 | C4 | SLAMF7 | C6 | - | - |
| CD24 | C4 | IGHV3-23 | C6 | - | - |
| FAM30A | C4 | IGHM | C6 | - | - |
| LY86 | C4 | IGLV2-14 | C6 | - | - |


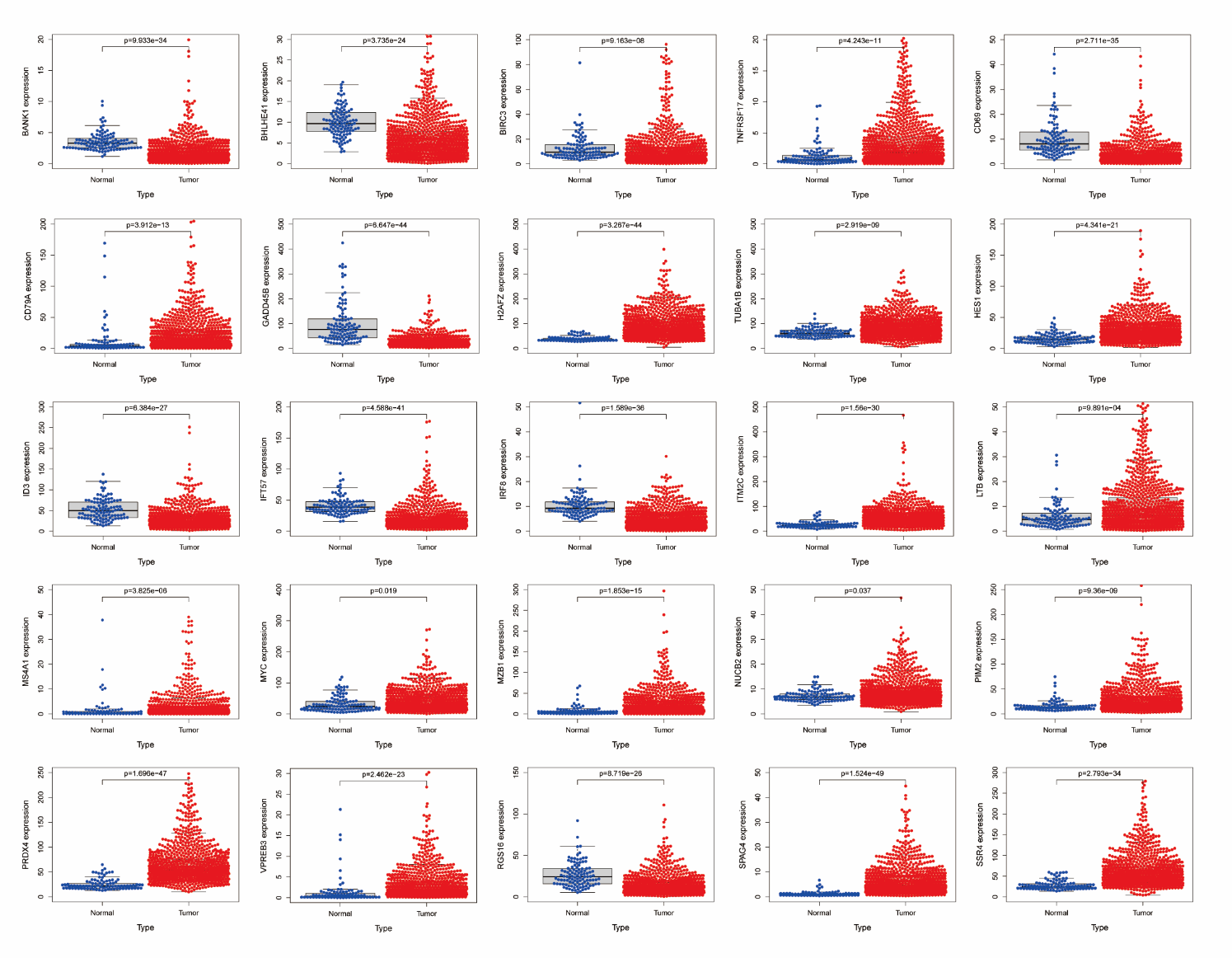
 **Supplementary Figure 1.** Comparison of the expression levels of B cell-related genes in BRGPs signature between NSCLC tumor and normal tissue. NSCLC, non-small cell lung cancer. BRGPs, B cell-related gene pairs.


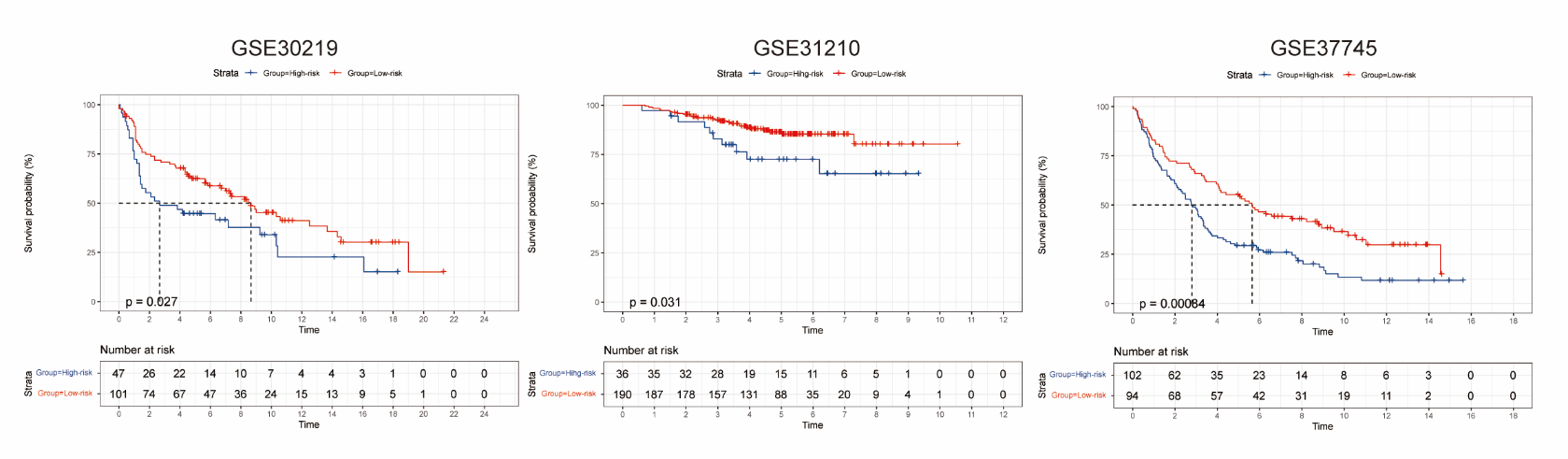


**Supplementary Figure 2.** Kaplan–Meier curves for patients with high-risk and low-risk scores in the additional GEO cohorts.


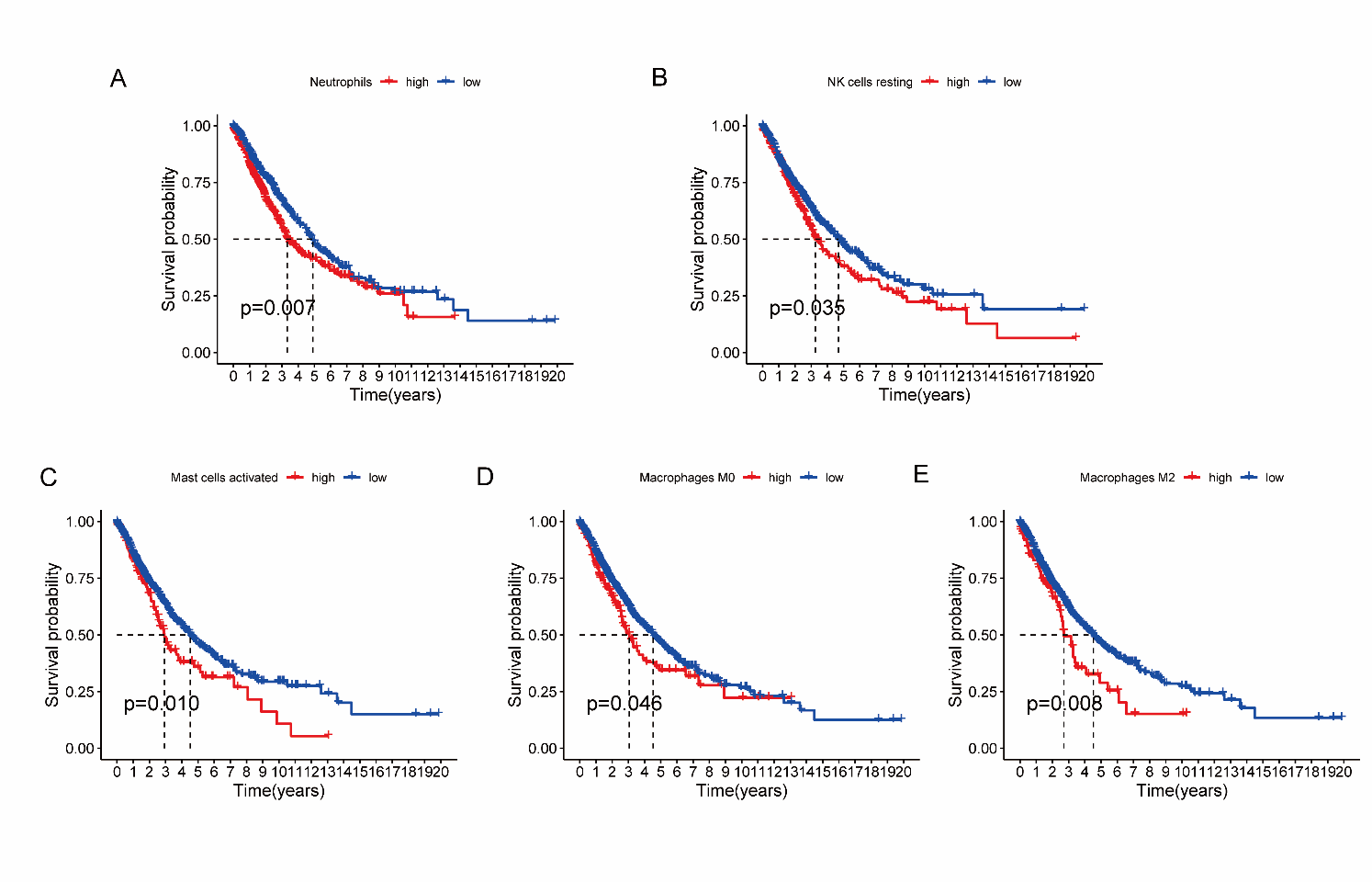


**Supplementary Figure 3.** Kaplan-Meier survival curves of overall survival in NSCLC patients. (A-E) Comparison of overall survival for NSCLC patients with different infiltration levels of neutrophils (A), resting NK cells (B), activated mast cells (C), M0 macrophages (D) and M2 macrophages (E) in the training cohort, respectively. NSCLC, non-small cell lung cancer.


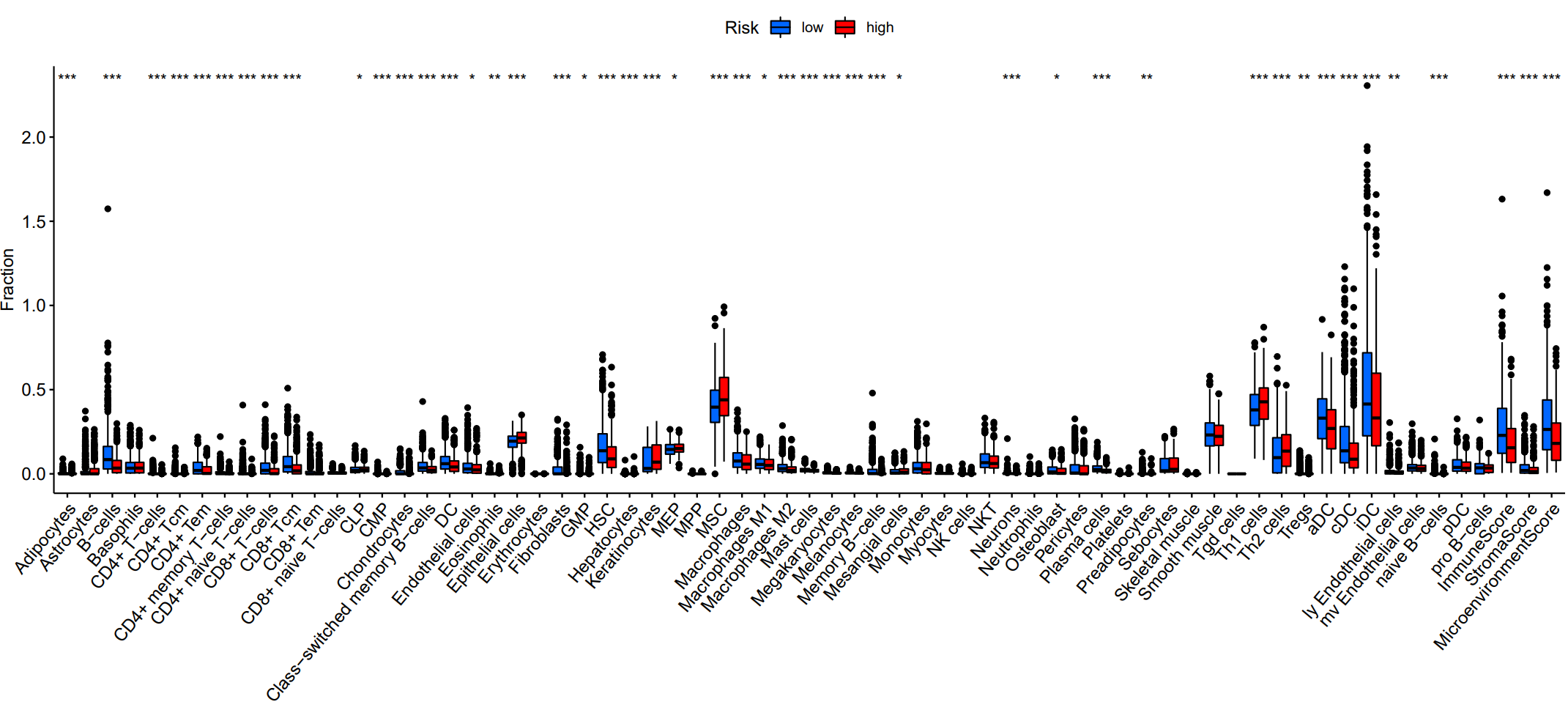
 **Supplementary Figure 4.** The fractions of different immune cells between high- and low-risk groups based on xCell method.


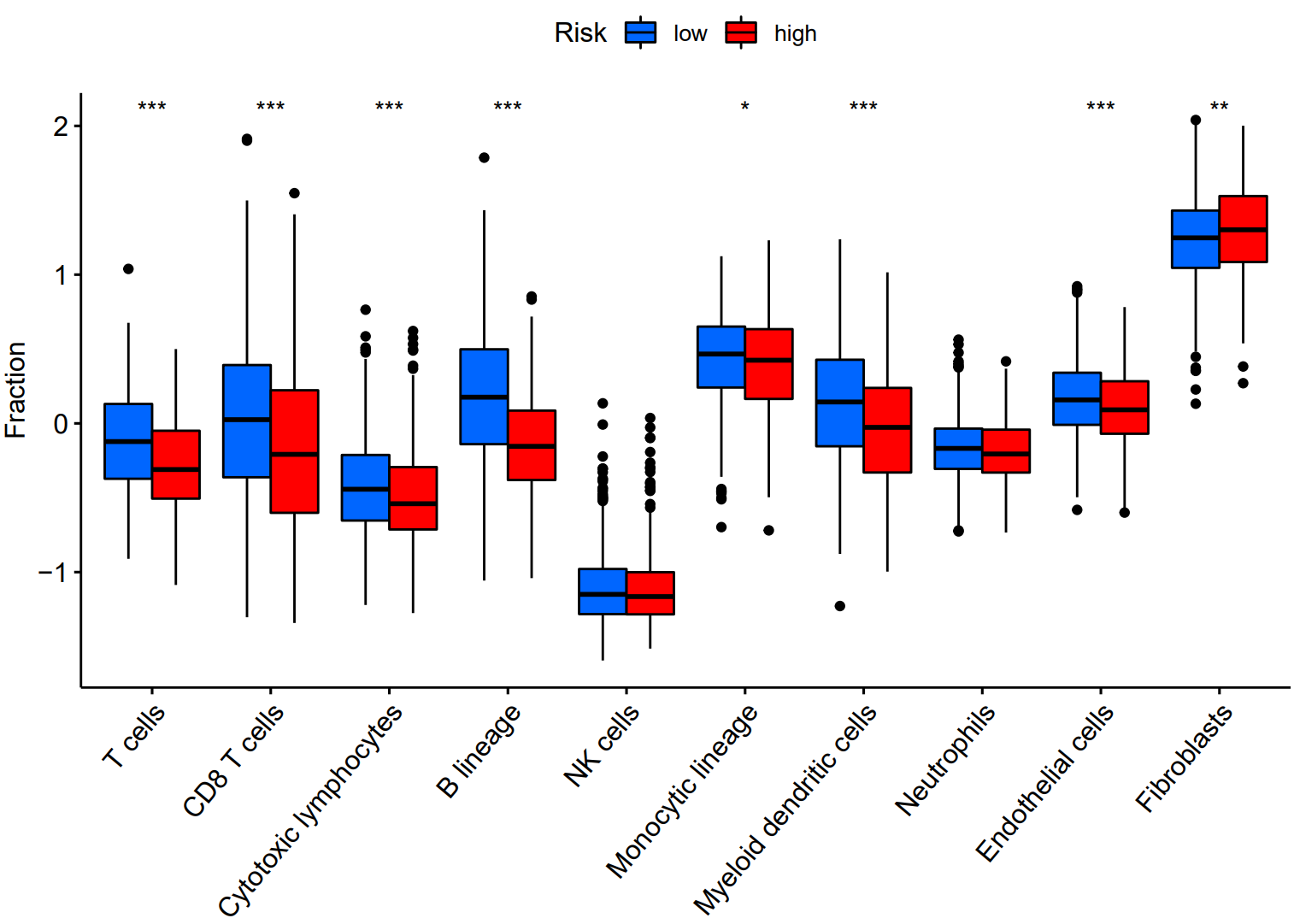
 **Supplementary Figure 5.** The fractions of different immune cells between high- and low-risk groups based on MCP-counter method.


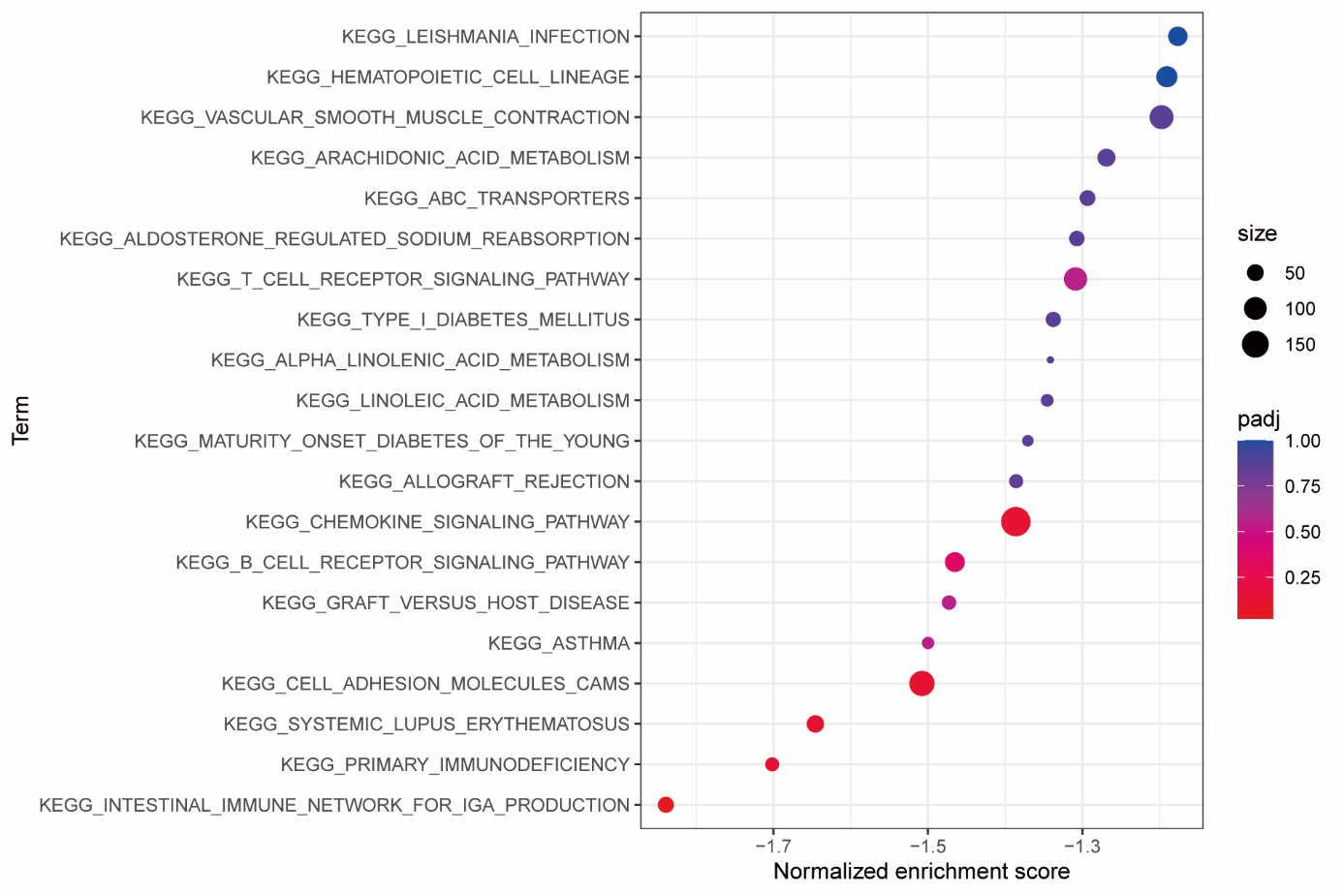


**Supplementary Figure 6.** Gene set enrichment analysis with KEGG pathway of low-risk BRGPs subgroup. KEGG, Kyoto Encyclopedia of Genes and Genomes. BRGPs, B cell-related gene pairs.


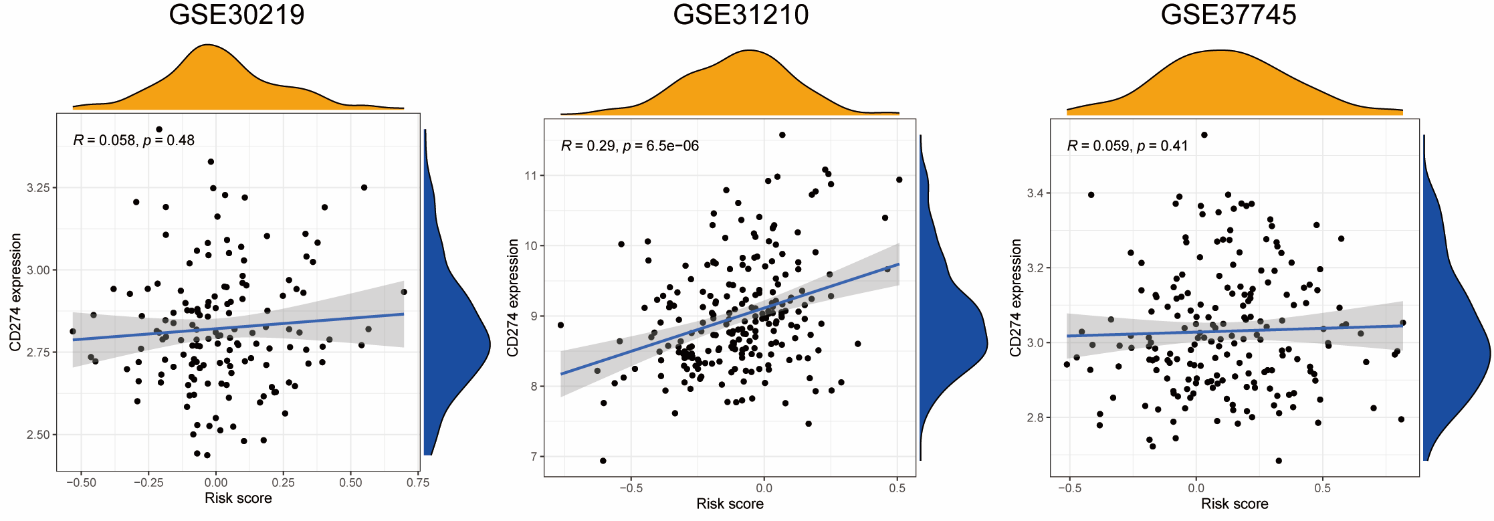


**Supplementary Figure 7.** The correlation analysis between the PD-L1 mRNA expression levels and the risk score in different GEO cohorts


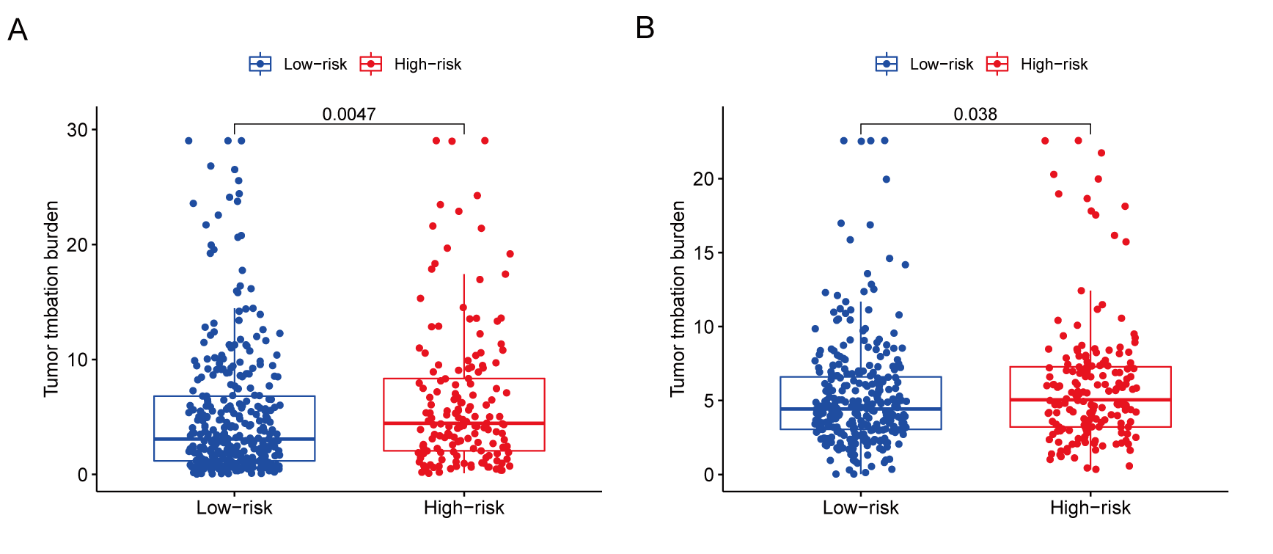


**Supplementary Figure 8.** TMB analysis in LUAD and LUSC patients. (A-B) The box plot shows the TMB scores between high- and low-risk groups in LUAD (A) and LUSC (B) patients. TMB, tumor mutational burden; LUAD, Lung adenocarcinoma; LUSC, lung squamous cell carcinoma.


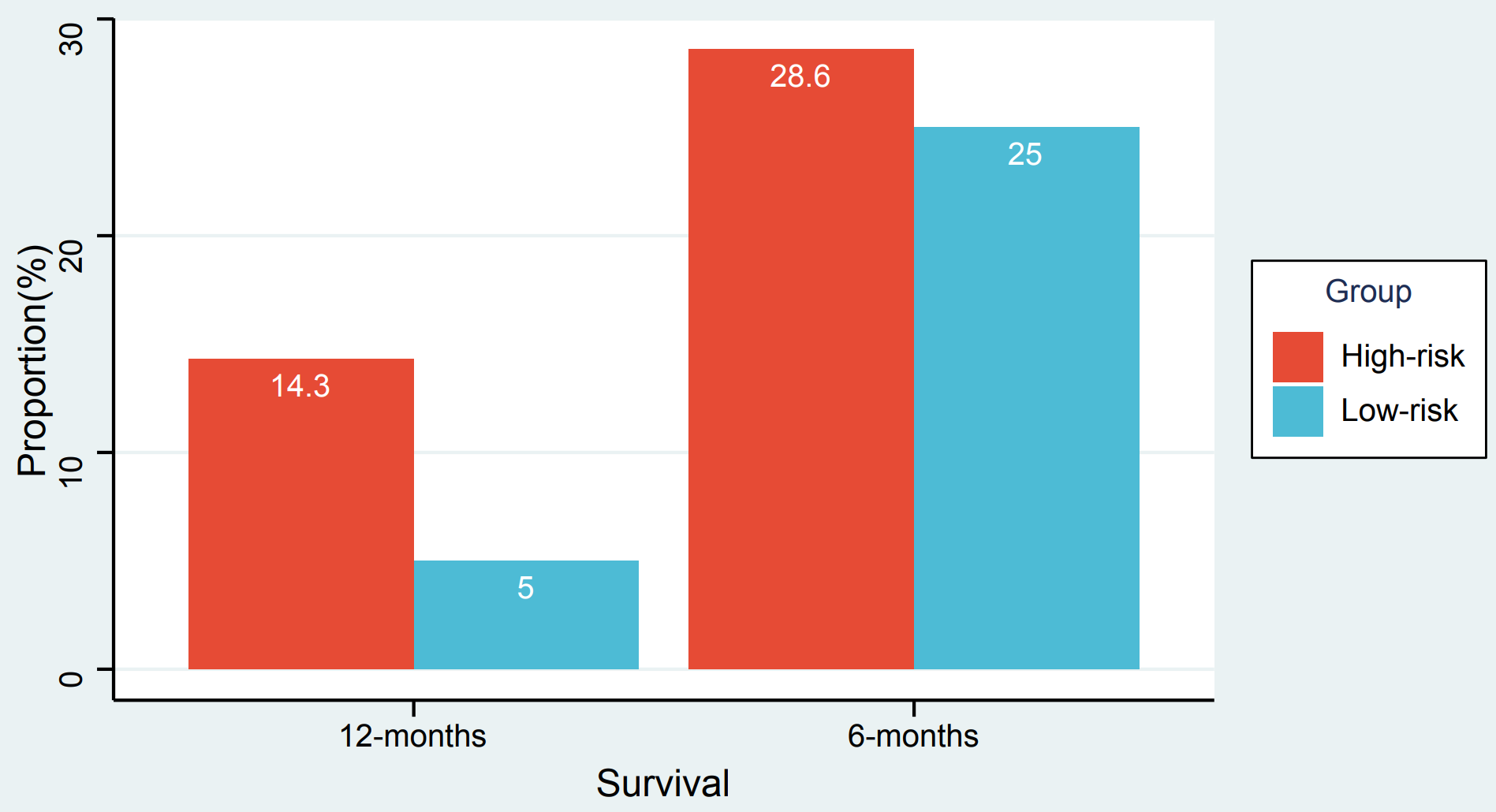


**Supplementary Figure 9.** 12- and 6-months survival rates of NSCLC patients with anti-PD-1 immunotherapy in high- and low-risk groups in the GSE135222 cohort.
